# Supplementary material for: Rapid Parallel Adaptation in Distinct Invasions of Ambrosia Artemisiifolia Is Driven by Large-Effect Structural Variants
Source: Mol Biol Evol. 2025 Jan 15;42(1):msae270. doi: 10.1093/molbev/msae270 (PMC11733498; doi:10.1093/molbev/msae270)
Supplement: msae270_Supplementary_Data [file msae270_supplementary_data.zip › RagweedAus_SFigs.pdf]

Supplementary Figures

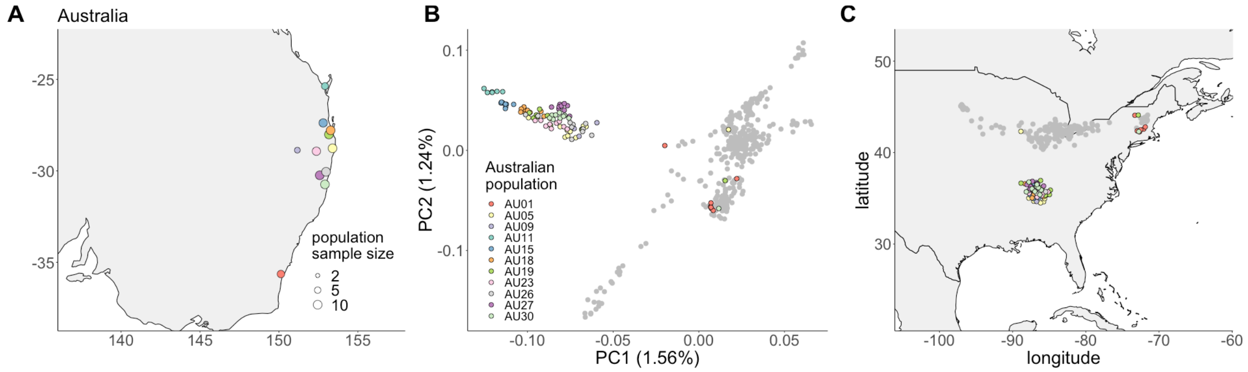

Figure S1. A. Geographic location of each Australian population. **B.** The first two principal components of putatively neutral genetic variation for each sample (as in Fig. 1B), with individual Australian samples coloured by population and North American and European samples in grey. **C**. Deep neural network-predicted source locations in North America for invasive-range samples (as in Fig. 1D) with European predictions in grey and Australian predictions coloured by population with colours corresponding to (A).

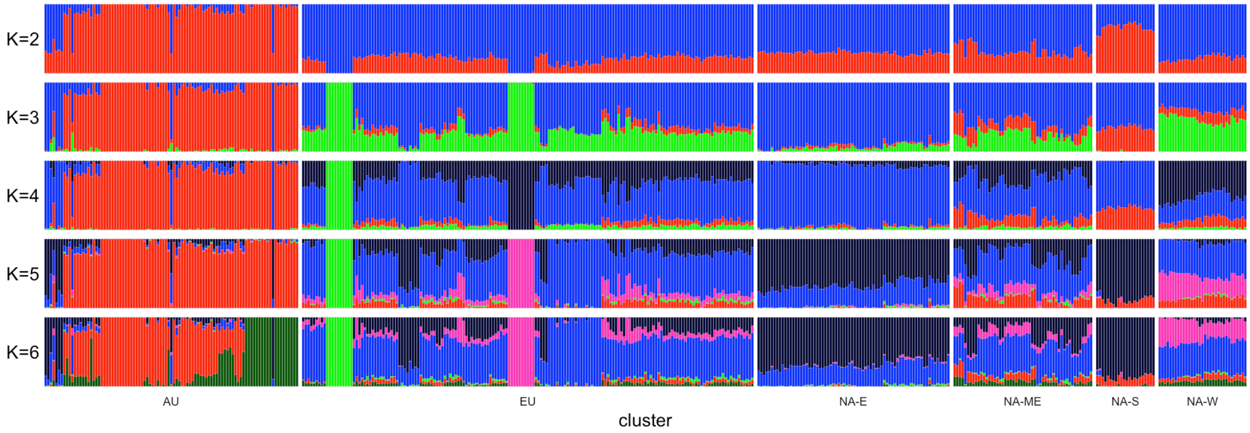

Figure S2. Admixture proportions for 443 *Ambrosia artemisiifolia* samples (vertical bars) for between two and six theoretical ancestral populations (*K*). North American and European samples are arranged by genetic-spatial clusters described in (Bieker et al. 2022). Within clusters samples are grouped by sampling location. AU: Australia; EU: Europe; NA-E: North America (east); NA-ME: North America (mid east); NA-S: North America (south); NA-W: North America (west).

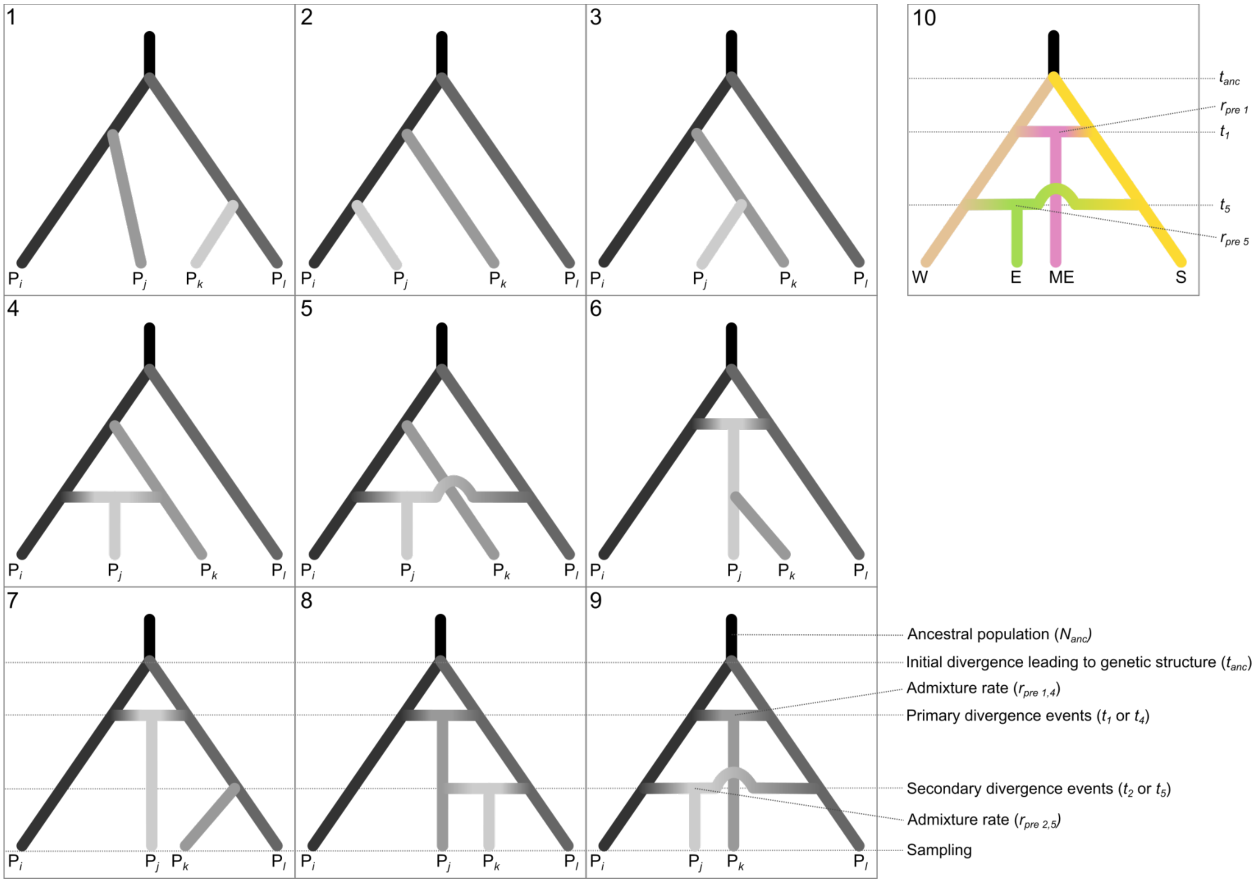

Figure S3. Graphical illustration of the development of genetic structure of *Ambrosia artemisiifolia* within its native range, tested using ABC-RF. For analyses 1-9, P_i, P_j, P_k, P_l represent the four sampled native range genetic units (W, ME, E, S). Scenarios within analyses 1-9 were generated by permuting the four genetic units at the branch tips for each of three divergence event timing configurations: both occurring early (t_1 and t_2); a combination of early and recent divergence events (t_1 and t_5), or; both occurring recently (t_4 and t_5). Effective population sizes, N_i, are represented in grayscale, with the 'ancestral population', N_{anc}, labeled and shown in black. For scenarios where divergence is due to one or more admixture events, two independent pre-introduction admixture rates ($rpre_{t1,t4}$, $rpre_{t2,t5}$) were modeled. The best-supported scenario from analysis 10–which compared well-supported scenarios from analyses 1-9–is shown in color. Time is not to scale. For detailed descriptions of parameters and prior intervals, refer to Table S13.

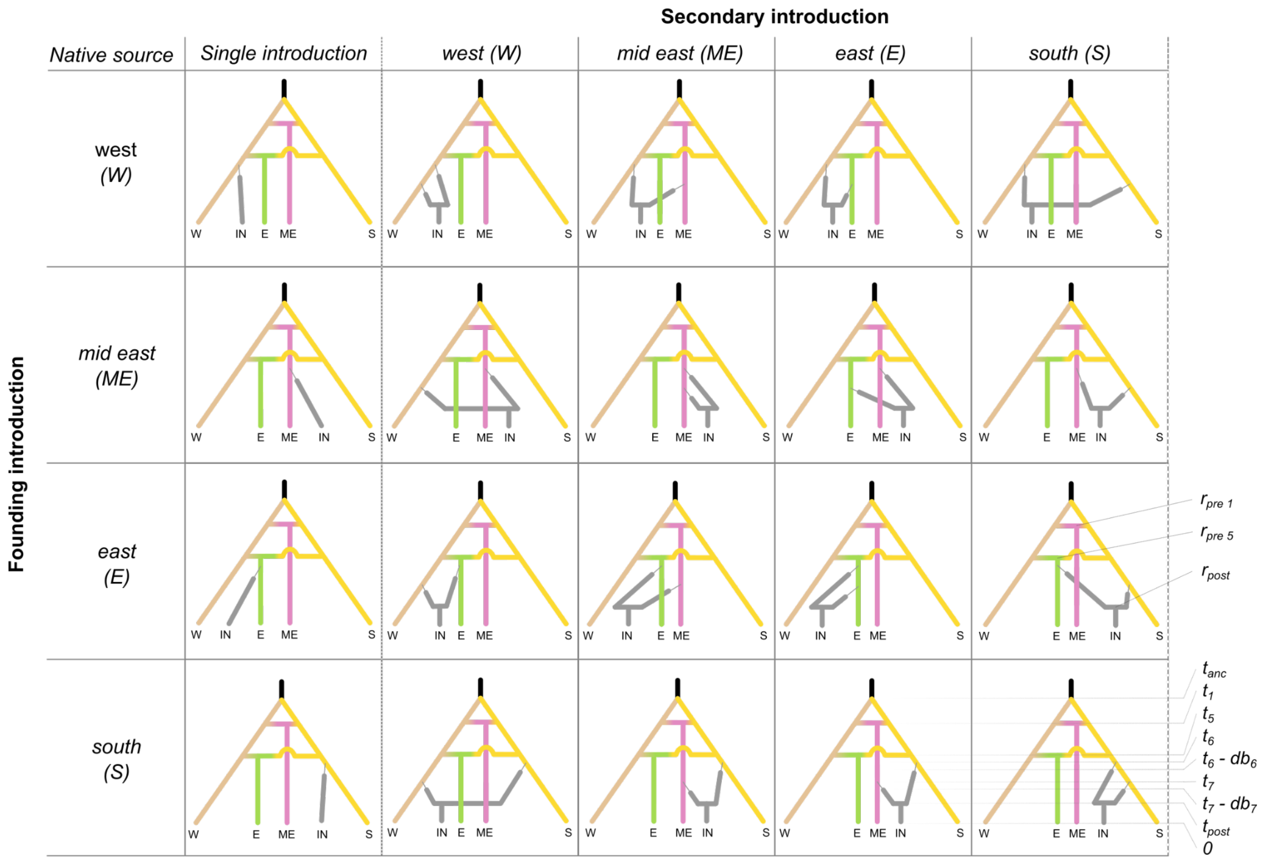

Figure S4. Graphical illustration of *Ambrosia artemisiifolia* introduction scenarios from divergent North American genetic units (W, E, ME, S) during initial (rows) and secondary (columns) introduction, tested using ABC-RF for European and Australian ranges (IN) independently.Thin lines indicate bottlenecks of duration db_i with effective population sizes of Nb_i. Time is not to scale. For detailed descriptions of parameters and prior intervals, refer to Table S13.

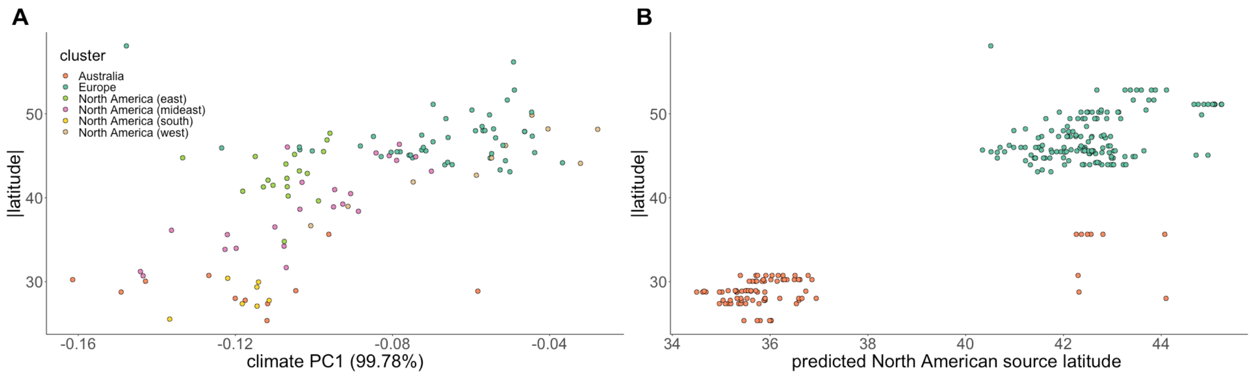

Figure S5. A. Relationships between population sampling locations in climate space (absolute latitude against the first principal component of four independent WorldClim variables [BIO1, BIO2, BIO12, BIO15]). **B.** Sampling location absolute latitude plotted against the *Locator*-predicted source location in North America for invasive-range samples.

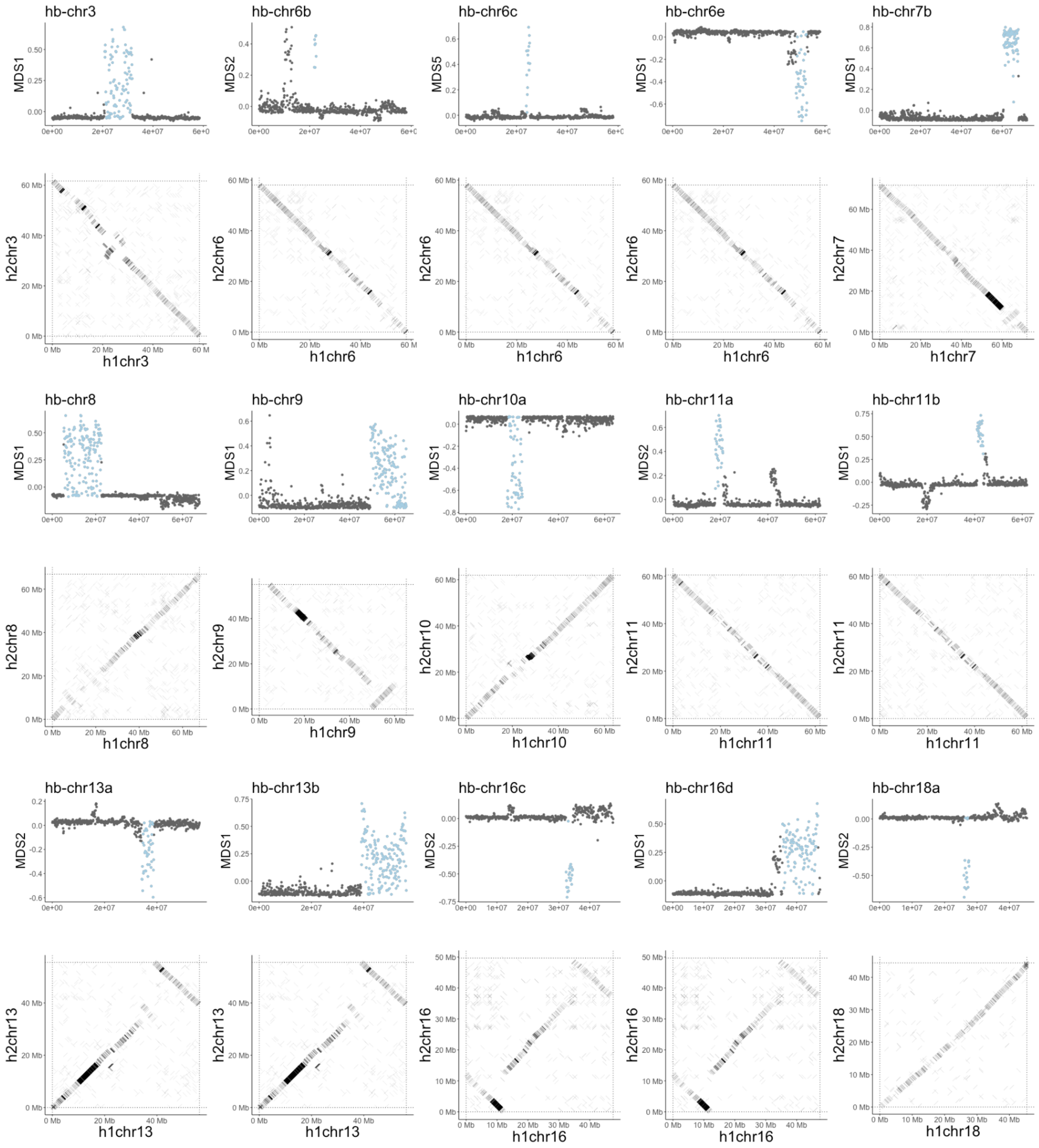

Figure S6. Divergent local population structure (MDS) in population-genomic data corresponds to inversion polymorphisms observed in alignments of homologous diploid reference chromosomes.

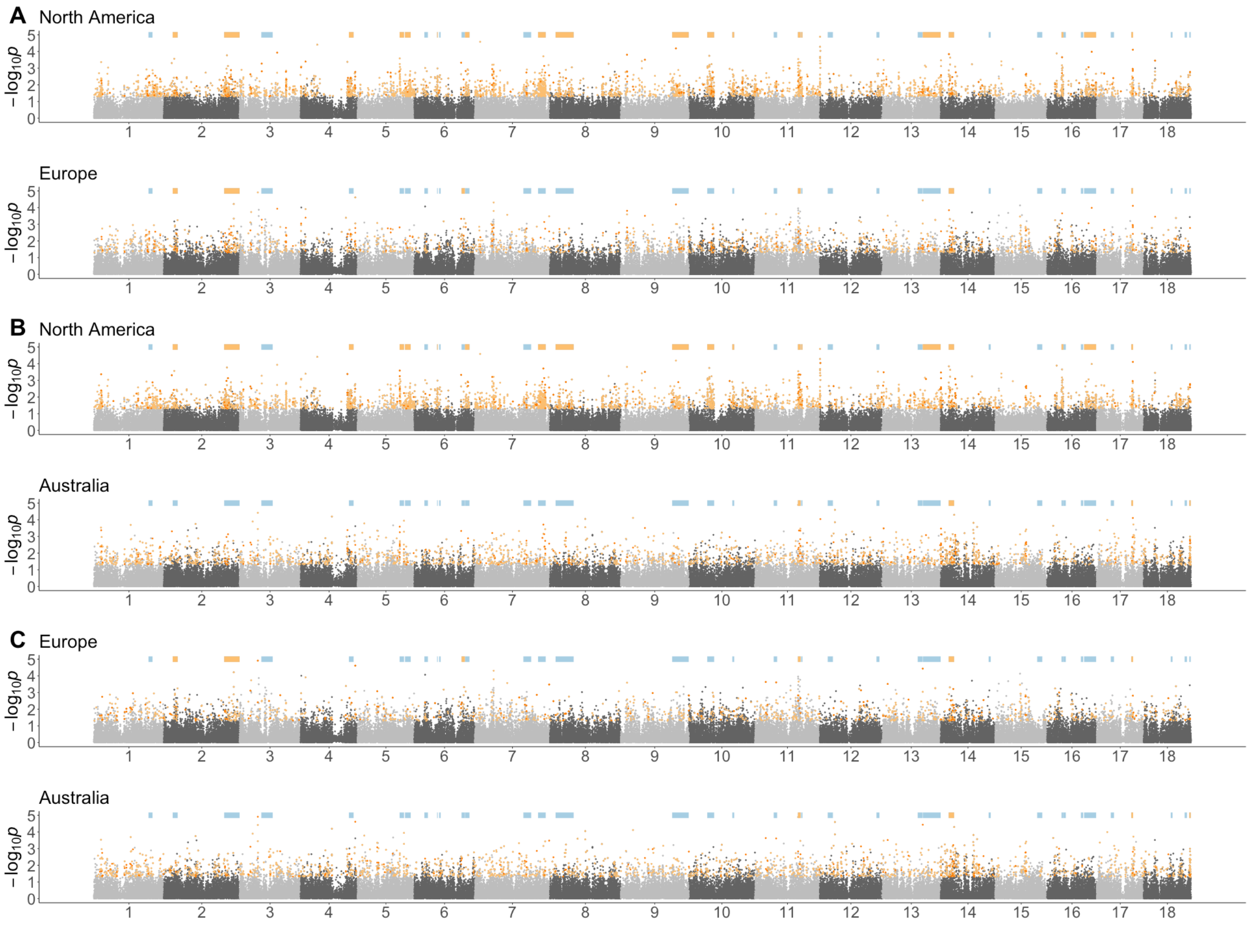

Figure S7. Manhattan plots (log-transformed empirical *p*-values for 10-kbp analysis window scores against genomic location) for XtX scans within each range separately. Orange highlights represent Xtx-EAA windows: the top 5% of XtX windows for each range that are also among the top 5% of EAA windows for at least one environmental variable in that range, with dark orange indicating outlier windows shared between each pairwise combination of ranges (**A**, **B** and **C**). Pale blue bars indicate the location of haploblocks. Orange bars denote haploblocks that are enriched for XtX-EAA windows.

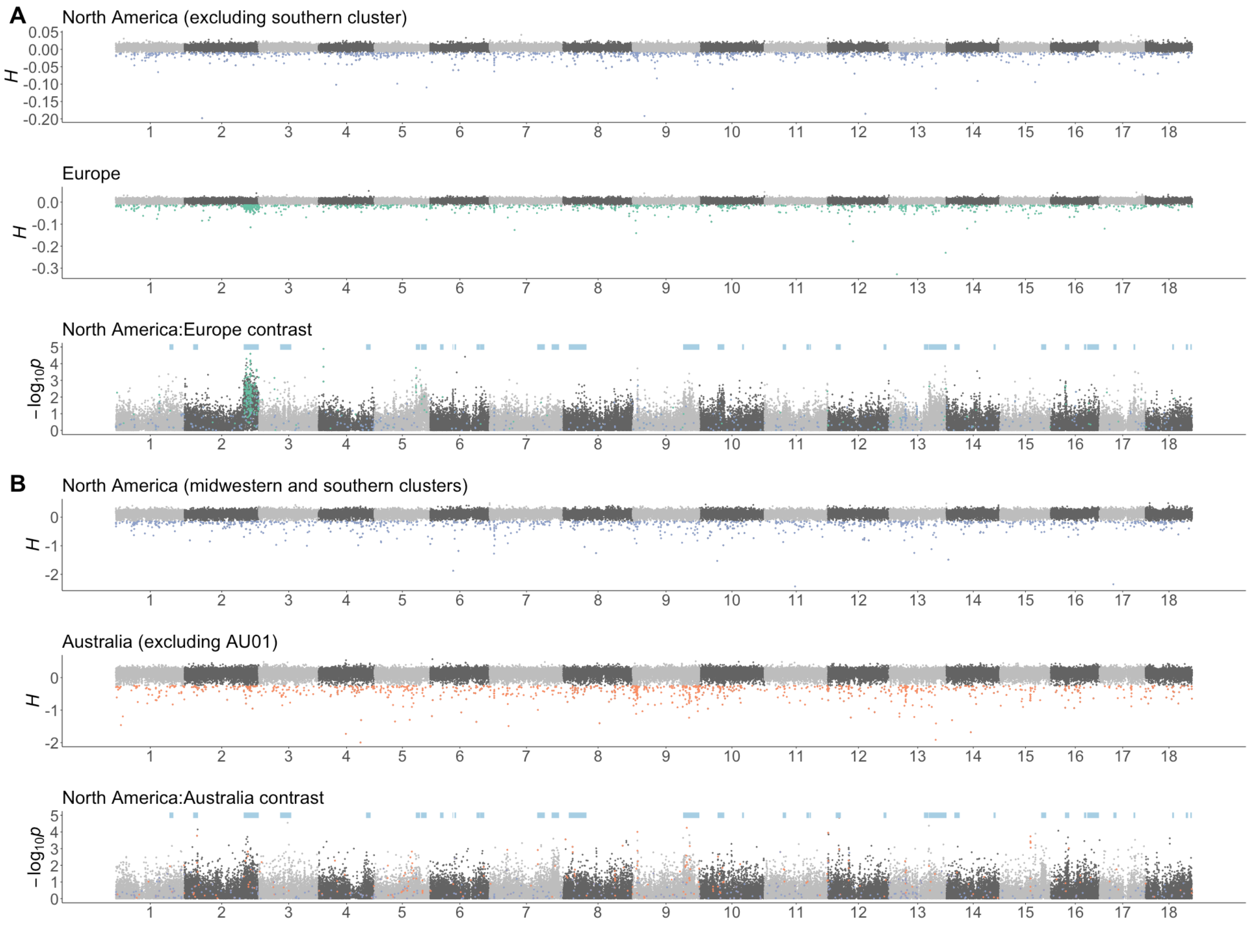

Figure S8. Manhattan plots for Fay and Wu's *H* in individual ranges, and contrast scans between ranges for each native-invasive pair (**A** and **B**). 1% *H* outliers are coloured (North America: blue; Europe: green; Australia: red) in each *H* plot as well as the corresponding contrast plot. Pale blue bars indicate the location of haploblocks.
