# Supplementary material for: Rapid Parallel Adaptation in Distinct Invasions of Ambrosia Artemisiifolia Is Driven by Large-Effect Structural Variants
Source: Mol Biol Evol. 2025 Jan 15;42(1):msae270. doi: 10.1093/molbev/msae270 (PMC11733498; doi:10.1093/molbev/msae270)
Supplement: msae270_Supplementary_Data [file msae270_supplementary_data.zip › RagweedAus_SMethods.pdf]

Supplementary Methods

Demographic modeling

We implemented an approximate Bayesian computation (ABC) random forest (RF) statistical framework using DIYABC-RF (Collin et al. 2021) to model the formation of genetic structure in the native range, infer the introduction source of the invaded ranges, and to estimate demographic parameters of *A. artemisiifolia*. In this framework, we simulated genetic data under various demographic scenarios based on user-defined priors (Table S13). We then compared summary statistics from these simulated data with those from the observed data (Beaumont et al. 2002; Beaumont 2010).

RF, a machine-learning algorithm, constructs decision trees from bootstrapped samples to perform classification tasks. These trees use summary statistics as predictor variables (Breiman 2001), allowing RF to identify the scenario that most closely aligns with the observed data. DIYABC-RF uses all available summary statistics (Table S15), supplemented with axes from linear discriminant analysis (LDA; Pudlo et al. 2016). The RF method reserves a portion of the simulated data as 'out-of-bag'. This data is used to estimate the error rate, providing a measure of accuracy comparable to using a test set of the same size as the training set (Pudlo et al. 2016).

We used the six genetic units. Four of these units represent different regions in the native North American range and were previously defined by Bieker et al. (2022) based on ADMIXTURE results and geography: (1) south (NA-S); (2) mid east (NA-ME); (3) east (NA-E); (4) western (NA-W). The remaining two genetic units were from the invaded ranges: (5) Europe (EU) and (6) Australia (AU). To account for potential unsampled sources of introductions, we included unsampled or 'ghost' genetic units (Slatkin 2005). These 'ghost' genetic units were used to define scenarios modeling multiple introductions, including repeated introductions from the same native source, leading to subsequent admixture in the introduced range.

We used all samples for which SNPs were called (*n*=391), except the southernmost Australian population, which appeared separate to the main genetic cluster (AU01; Fig. S1). All SNPs with MAF>0.05 were included in the analysis. Using plink 2.0 alpha (Chang et al. 2015), all SNPs were filtered to include only those that were LD pruned (`--indep-pairwise 50 5 0.2`) and polymorphic (`--geno 1`). Resulting filtered VCF files were converted to '.snp' format suitable for DIYABC-RF analysis using a custom script.

We extended the ABC-RF analysis by van Boheemen et al. (2017), incorporating independent introduction and bridgehead introduction scenarios for each invaded range. These scenarios included founding, secondary, and bottlenecked introductions. We modeled admixture between genetic units both before and after introduction. For the introduction scenarios, we used the priors defined in van Boheemen et al. (2017; Table S13).

We first modeled the divergence of the genetic clusters in the native range. Due to uncertainty around the values of historical priors, we adjusted the lower and upper prior limits based on the prior distributions of the preliminary simulated datasets to ensure a reasonable compatibility between the observed and simulated datasets (Collin et al. 2021). To achieve this we used DIYABC-RF's visual and numerical outputs, including the projection of the datasets on the first linear discriminant analysis (LDA) axes and the proportion of simulated data with summary statistics values below those of the observed dataset (Collin et al. 2021). This allowed us to set the prior distribution as wide as possible, accommodating our uncertainty, while remaining within biological reason (Bertorelle et al. 2010).

To model the formation of genetic structure in the native range of *A. artemisiifolia*, we simulated datasets from nine branching pattern topologies (Fig. S3). Each topology contains three temporal parameters (t_{anc}, t_1 or t_4, t_2 or t_5), within the prior interval of which branching events could occur by divergence or admixture (Table S2). These parameters were configured in three ways: all branching occurring early (t_1 and t_2), a combination of early and recent branching (t_1 and t_4), or all branching events occurring recently (t_4 and t_5). For each topology we considered all configurations of temporal parameters, and all permutations of the four sampled native range populations. This resulted in the generation of 648 unique scenarios (9 topologies × 3 configurations × 4! permutations).

To manage the number of native range scenarios to be compared with ABC-RF we opted for a sequential approach to model selection (Table S2; Table S3; Table S14; Byrne et al. 2022). Initial model choice analyses were conducted on scenarios within their respective topologies (Table S2, analyses 1-9). Scenarios that achieved an above-average number of classification votes (i.e., no. RF trees / no. scenarios) within their topology were then competed across topologies (Table S2, analysis 10). This ensured that well-supported scenarios were directly compared. To determine model choice consistency, we replicated these analyses with

independent simulated datasets, using a larger subset of available SNPs and a higher number or RF classification trees (Chapuis et al. 2020).

We conducted independent, stepwise analyses of the introduction of *A. artemisiifolia* to Europe and Australia, incorporating the results of the preceding native range analysis (Fig. S4; Table S3). The native range scenario with the highest posterior probability from the across-topology native range analysis was used as the ancestral scenario for the primary and/or secondary introductions (Fontaine et al. 2021).

We compared scenarios of independent and bridgehead introductions by generating 64 combination scenarios from independent introduction scenarios that received above-average classification votes (Table S3). These were compared against 8 scenarios consisting of above-average independent European introduction scenarios given a bridgehead introduction to Australia (Table S14). Model choice analysis was conducted with an increasing training set (starting at 5,000 per scenario) and trees (starting at 30,000) until model choice converged for eight consecutive runs. The proportion of votes, global prior error rate and posterior probability values are averaged over the replicates.

To assess if scenario choice was sensitive to the number of bridgehead vs. non-bridgehead scenarios, we conducted model choice analysis on a subset of scenarios. In this analysis, the scenario for the non-bridgehead introduction to Australia was based on the scenario with the highest posterior probability from the independent Australian introduction analysis. This scenario was combined with the 8 above-average European introduction scenarios and then competed against an equal number of bridgehead introduction scenarios.

References

Beaumont MA. 2010. Approximate Bayesian computation in evolution and ecology. *Annual review of ecology, evolution, and systematics* 41:379–406.

Beaumont MA, Zhang W, Balding DJ. 2002. Approximate Bayesian computation in population genetics. *Genetics* 162:2025–2035.

Bertorelle G, Benazzo A, Mona S. 2010. ABC as a flexible framework to estimate demography over space and time: some cons, many pros: THE ABC REVOLUTION IN NINE STEPS. *Mol. Ecol.* 19:2609–2625.

Bieker VC, Battlay P, Petersen B, Sun X, Wilson J, Brealey JC, Bretagnolle F, Nurkowski K, Lee C, Barreiro FS, et al. 2022. Uncovering the genomic basis of an extraordinary plant invasion. *Sci Adv* 8:eabo5115.

van Boheemen LA, Lombaert E, Nurkowski KA, Gauffre B, Rieseberg LH, Hodgins KA. 2017. Multiple introductions, admixture and bridgehead invasion characterize the introduction history of Ambrosia artemisiifolia in Europe and Australia. *Mol. Ecol.* 26:5421–5434.

Breiman L. 2001. Random forests. *Mach. Learn.* 45:5–32.

Byrne D, Scheben A, Scott JK, Webber BL, Batchelor KL, Severn-Ellis AA, Gooden B, Bell KL. 2022. Genomics reveals the history of a complex plant invasion and improves the management of a biological invasion from the South African-Australian biotic exchange. *Ecol. Evol.* 12:e9179.

Chang CC, Chow CC, Tellier LC, Vattikuti S, Purcell SM, Lee JJ. 2015. Second-generation PLINK: rising to the challenge of larger and richer datasets. *GigaScience* [Internet] 4. Available from: http://dx.doi.org/10.1186/s13742-015-0047-8

Chapuis M-P, Raynal L, Plantamp C, Meynard CN, Blondin L, Marin J-M, Estoup A. 2020. A young age of subspecific divergence in the desert locust inferred by ABC random forest. *Mol. Ecol.* 29:4542–4558.

Collin F-D, Durif G, Raynal L, Lombaert E, Gautier M, Vitalis R, Marin J-M, Estoup A. 2021. Extending approximate Bayesian computation with supervised machine learning to infer demographic history from genetic polymorphisms using DIYABC Random Forest. *Mol. Ecol. Resour.* 21:2598–2613.

Fontaine MC, Labbé F, Dussert Y, Delière L, Richart-Cervera S, Giraud T, Delmotte F. 2021. Europe as a bridgehead in the worldwide invasion history of grapevine downy mildew, Plasmopara viticola. *Curr. Biol.* 31:2155–2166.e4.

Pudlo P, Marin JM, Estoup A, Cornuet JM, Gautier M, Robert CP. 2016. Reliable ABC model choice via random forests. *Bioinformatics* 32:859–866.

Slatkin M. 2005. Seeing ghosts: the effect of unsampled populations on migration rates estimated for sampled populations: GHOST POPULATIONS. *Mol. Ecol.* 14:67–73.
